# Assessing the Application and Effectiveness of Human Amniotic Membrane in the Management of Venous and Diabetic Ulcers: A Systematic Review and Meta-Analysis of Randomized Controlled Trials

**DOI:** 10.7759/cureus.56659

**Published:** 2024-03-21

**Authors:** Abeer A Alomairi, Rama A Alhatlani, Shahad M Alharbi, Zainab J Alqurain, Ibtihal Z Alanazi, Sarah A Alanazi, Zainab Alkhmis, Alaa F Samandar, Sultan Arif

**Affiliations:** 1 College of Medicine, King Saud Bin Abdulaziz University for Health Sciences, Riyadh, SAU; 2 College of Medicine, Unaizah College of Medicine and Medical Sciences, Qassim University, Qassim, SAU; 3 College of Medicine, King Abdulaziz University, Jeddah, SAU; 4 College of Medicine, Batterjee Medical College, Jeddah, SAU; 5 Medicine, Northern Border University, Arar, SAU; 6 Medicine and Surgery, King Faisal University, Alhasa, SAU; 7 College of Medicine, Umm AlQura University - Al-Qunfudah Branch, Mecca, SAU; 8 Department of Plastic Surgery and Burn Unit, Security Force Hospital, Riyadh, SAU

**Keywords:** diabetic foot ulcers, venous ulcers, leg ulcers, venous, amniotic membrane, human amniotic membrane

## Abstract

This study aimed to assess the efficacy of human amniotic membranes (HAM) in treating venous and diabetic ulcers, which often pose challenges in healing. A systematic review and meta-analysis were conducted, evaluating 10 relevant studies involving 633 participants. Findings revealed that HAM treatment significantly accelerated ulcer closure, demonstrating over 90% complete healing compared to standard care. Despite moderate heterogeneity among studies, the results strongly suggested the effectiveness and safety of HAM therapy for venous and diabetic leg ulcers. Further research with larger study cohorts is recommended to bolster the existing evidence supporting HAM in managing these challenging wounds.

## Introduction and background

Chronic wounds, including diabetic and venous leg ulcers, represent a significant healthcare challenge worldwide due to their high prevalence, prolonged healing times, and potential for complications. The management of these wounds often requires advanced therapeutic interventions to promote optimal healing and prevent further deterioration. In recent years, human amniotic membrane (HAM) has emerged as a promising regenerative therapy for chronic wounds, offering unique biological properties that can enhance the wound healing process [1]. 

Diabetic ulcers, a common complication of diabetes, are characterized by impaired wound healing and increased susceptibility to infections [2]. Venous leg ulcers (VLU), on the other hand, often result from venous insufficiency and chronic inflammation, leading to non-healing wounds that significantly impact patients' quality of life [3]. The etiology of venous leg ulcers arises from a combination of venous hypertension and venous insufficiency. Venous hypertension refers to the elevated pressure within the veins, typically occurring in the lower extremities. Venous insufficiency, on the other hand, is a condition where the venous valves fail to adequately prevent blood from flowing backward, leading to the pooling of blood in the veins [4]. Conventional treatment approaches for these chronic wounds include wound debridement, compression therapy, and topical dressings. However, a subset of patients may exhibit slow or inadequate responses to standard treatments, necessitating the exploration of alternative therapeutic modalities such as HAM [1].

The human amniotic membrane, derived from the innermost layer of the placenta, possesses unique properties that make it an attractive candidate for wound healing applications. It is rich in extracellular matrix proteins, growth factors, and cytokines. These components play a crucial role in wound healing processes. They have the ability to induce angiogenesis (formation of new blood vessels) and promote dermal fibroblast proliferation (cells responsible for tissue repair and collagen production), and attract mesenchymal stem cells that are vital for wound repair and regeneration. This makes the amniotic membrane a valuable resource in medical applications related to wound healing and tissue regeneration [5].

On the other hand, another mechanism through which the amniotic membrane facilitates the wound healing process is by accelerating the regenerative processes of damaged tissue via the delivery of hyaluronan polymers. Hyaluronan, a naturally occurring polysaccharide, plays a crucial role in tissue regeneration and repair, and its controlled release by the amniotic membrane aids in promoting the efficient healing of wounds. Therefore, the selection of an appropriate amniotic tissue processing method is crucial in order to preserve tissue quality, maintain tissue integrity, and retain the inherent biological properties. Amniotic tissues possess unique biological properties that make them effective in promoting the healing process of ulcers caused by various factors, including burns, diabetes, neuropathy, and bedsores [6].

Another study shows that the immunogenicity of human amnion/chorion allografts is notably low, with rare instances of rejection reported. Furthermore, histological analysis and histopathology biopsy studies conducted on ulcers treated with HAM consistently revealed favorable outcomes. These include a substantial increase in granulation tissue formation, enhanced deposition of connective tissue, notable development of the basement membrane, and improved vascularization. In addition to its aforementioned benefits, HAM possesses various other properties, including antifibrotic, anti-inflammatory, angiogenic, bacteriostatic, and thrombolytic properties. Furthermore, studies have documented that HAM transfers induce changes in thick-walled vessels, resulting in a reversion towards normal vasculature characterized by vessel wall thinning and widening of the lumen [6].

Furthermore, another study was done by Bailo et al. The summary of their findings suggests that amnion and chorion cells possess inherent advantages as a source of progenitor cells. Their research indicates that these cells hold significant potential for diverse applications in the field of cell therapy and transplantation procedures [7].

In their groundbreaking research, Toda et al. made a significant breakthrough by demonstrating the pluripotency of amnion-derived cells, which have the remarkable ability to differentiate into all three germ layers both in vitro and in vivo. This discovery not only underscores the developmental potential of these cells but also highlights their unique capacity to integrate into surrounding tissue and effectively respond to a degraded environment. Consequently, these findings have far-reaching implications, suggesting that amnion-derived cells hold tremendous therapeutic potential for a diverse range of diseases [8]. 

This systematic review and meta-analysis aim to critically assess the application and effectiveness of HAM in the treatment of diabetic and venous leg ulcers through the reviewing of randomized controlled trials (RCTs). By synthesizing the existing evidence, this study seeks to provide valuable insights into the clinical utility of HAM and its potential impact on improving patient outcomes in the management of chronic wounds.

## Review

Methods and materials

Registration and Literature Search Strategy

This systematic review was prospectively registered in The International Prospective Register of Systematic Reviews (PROSPERO) (ID number: CRD42023441194) and conducted in adherence to the Preferred Reporting Items for Systematic Reviews and Meta-Analysis (PRISMA) guidelines. A comprehensive electronic search was conducted of Ovid MEDLINE, Google Scholar, Embase, and Cochrane Library databases for studies published from inception to June 2023. The search strategy was designed independently by one author and approved by the rest of the team. A combination of Medical Subject Headings (MeSH) terms such as “human amniotic membrane” OR “amniotic membrane” AND “venous leg ulcers” OR “diabetic leg ulcers” was used to comprehensively identify all studies assessing the application and effectiveness of human amniotic membrane for managing venous leg ulcers. References of selected studies were further reviewed to identify any missing articles.

Study Selection

We included studies that met the following inclusion criteria: (1) RCTs; (2) participants aged 18 years or older with venous and diabetic ulcers; (3) studies published in English; (4) studies comparing amniotic membrane to standard of care treatment for venous and diabetic ulcers; (5) reporting one or more outcomes including healing rate, healing time, percent area reduction, infection rate, or other adverse events. 

Studies were excluded if they: (1) included patients under 18 years old; (2) were duplicate studies; (3) were non-RCT study designs such as reviews, case reports, cohort studies, retrospective studies, or animal experiments; (4) did not use human amniotic membrane to treat venous and diabetic ulcers; (5) had high risk of bias or low quality based on assessment of study design, sample size, data collection/analysis, and other relevant factors.

**Figure 1:**
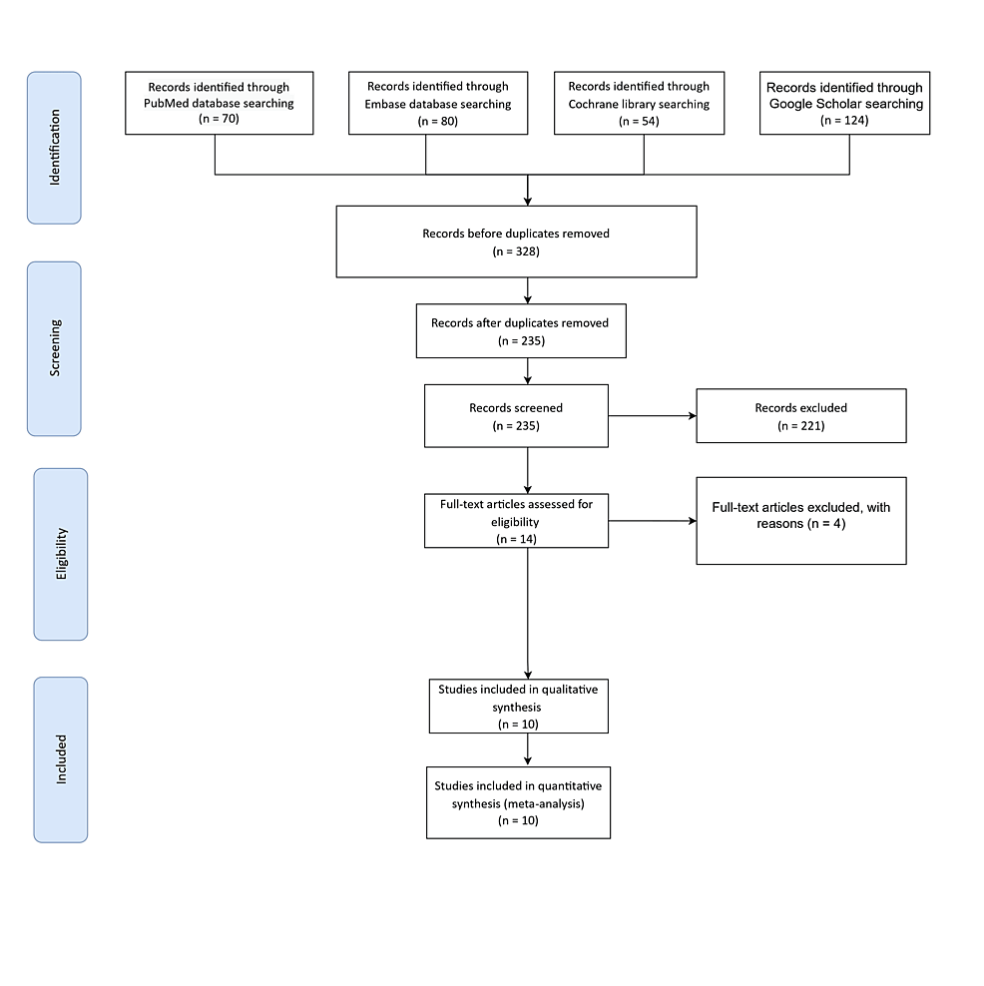
The flowchart of the reviewed studies according to Preferred Reporting Items for Systematic Reviews and Meta-Analysis (PRISMA).

All records from the primary search were imported to RAYYAN for deduplication and screened by three authors for relevance based on title and abstract. Full texts of retained studies were independently screened by four authors for final inclusion/exclusion. Any disagreements during screening were resolved through discussion and consensus amongst all authors. The PRISMA flow diagram of study selection is shown in Figure 1.

Data Extraction

Four authors independently extracted data from the included studies using a standardized data extraction sheet. Extracted information included year and country of publication, journal name, study design, sample size, type of amniotic membrane used, ulcer etiology and size, comorbidities, complications, interventions, and outcomes.

Risk of Bias Assessment

Two reviewers independently assessed the risk of bias in the eligible RCTs using the revised Cochrane risk-of-bias tool (RoB 2). Each RCT was categorized as having "high risk," "low risk," or "some concerns" of bias, as shown in Table 1. Any discrepancies were resolved through discussion until a consensus was reached.

**Table 1:** Review authors' judgments about each risk of bias (RoB) item for each included study.

	Bias arising from the randomization process	Bias due to deviations from intended interventions	Bias due to missing outcome data	Bias in measurement of the outcome	Bias in selection of the reported result	Overall RoB
Serena TE et al., 2022 [9]	Low	Low	Low	Low	Low	Low
Serena TE et al., 2020 [10]	Low	Low	Low	Low	Low	Low
Snyder RJ et al., 2016 [11]	Low	Low	Low	Low	Low	Low
Bianchi C et al., 2018 [12]	Low	Low	Low	Low	Low	Low
DiDomenico LA et al., 2016 [13]	Low	Low	Low	High	Low	Low
Lavery LA et al., 2014 [14]	Low	Low	Low	Low	Low	Low
Zelen CM et al., 2014 [15]	Low	Low	Low	High	Low	Low
TetteIbach et al., 2019 [16]	Low	high	Low	Low	Low	Low
Zelen CM et al., 2013 [17]	Low	high	Low	Low	Low	Low
Zelen CM et al., 2016 [18]	Low	high	Low	Low	Low	Low

The Cochrane RoB 2 tool evaluates study quality across five domains using signaling questions to assess the risk of bias. Judgments for each domain can be ‘low risk’ (if low risk for all domains), ‘some concerns’ (if some concerns in at least one domain), or ‘high risk’ (if high risk in at least one domain or some concerns in multiple domains). Two authors conducted the risk of bias assessment independently, with disagreements resolved through consensus after consulting the senior author. We also evaluated all included articles according to the American Society of Plastic Surgeons’ levels of evidence and grading recommendations, as presented in Table 1.

Data Synthesis

Data analysis was performed using RevMan 5.3 software endorsed by the Cochrane Collaboration. All analyses used a random-effects model. The significance level was set at 95% confidence interval with p≤0.05. Statistical heterogeneity was assessed using the I 2 statistic and chi-square test p-values. For binary outcomes, data were pooled as risk ratios (RR) with 95% CIs. For continuous outcomes, mean differences (MD) or standard mean differences (SMD) were used.

Results

Characteristics of Included Studies and Patient Profile

The initial search yielded a total of 328 articles, distributed across different databases as follows: 70 articles were retrieved from MEDLINE, 80 from EMBASE, 124 from Google Scholar, and 54 from the Cochrane Library. Upon removal of duplicates, a total of 235 unique studies remained, whose titles and abstracts underwent thorough screening. Following a comprehensive evaluation of the full-text versions of 14 articles, a final selection of 10 articles was made, based on meticulous adherence to the predefined inclusion and exclusion criteria [9-18]. The excluded articles were omitted for various reasons, inadequate data availability in one, and non-randomized controlled trial designs in three. Notably, the timeframe for the publication of the studies encompassed the period from inception up until June 2023.

A comprehensive overview of the studies detailing the effects of amniotic membrane in the management of venous leg ulcers is provided in Table 2. It is important to underscore that all the studies incorporated into this review strictly adhered to the randomized controlled trial format, and notably, they were all conducted within the United States. For a visual representation of the systematic review process in accordance with the PRISMA guidelines, refer to Figure 1.

**Table 2:** Characteristics of the studies included. *Data presented as mean, standard deviation or number (%) as indicated. M; Male, F; Female, *; They didn’t specify if male or female, RCT; Randomized Control Trial. HAM; human amniotic membrane

Author	Year	Country	Study Design	Sex		Age in Year, Mean (SD)		Ulcer Size, Mean (SD)		Positive Results (% of healing)	
				HAM used	HAM not used	HAM used	HAM not used	HAM used	HAM not used	HAM used	HAM not used
E. Serena et al. [9]	2022	USA	Multicenter RCT	M: 19 - F: 21	M:13 - F: 7	70.0 (15.6)	70.0 (12)	4.7 (2.9)	6.0 (4.2)	45% of subjects achieve complete wound healing	0% of subjects achieve complete wound healing
E. Serena et al. [10]	2020	USA	RCT	M: 30 - F: 8	M: 29 - F: 9	59.2 (7.61)	59.6 (10.72)	3.33 (4.62)	3.12 (3.86)	60% complete healing	35% complete healing
J. Snyder et al. [11]	2016	USA	RCT	M : 11 - F: NA	M: 10 - F: NA	57.9 (12.49) Range: 34-85	58.6 (6.97) Range: 48-71.2	6.9	4.7	At 12 weeks = 81%	At 12 weeks = 55%
Bianchi et al. [12]	2018	USA	Multicenter RCT	M: 33 - F: 19	M: 39 - F:18	61.5 (14.9)	60.0 (10.6)	8.3 (6.7)	7.6 (6.1)	At 6 weeks = 77%	At 6 weeks = 8%
DiDomenico et al. [13]	2016	USA	RCT	M: 11 - F: 9	M: 16 - F: 4	59	58	3.3	2	At 12 weeks = 97%	At 12 weeks = 73%
Lavery et al. [14]	2014	USA	Multicenter, single-blinded RCT	M: 33 - F: 17	M: 35 - F: 12	55.5 (11.5)	55.1 (12.0)	3.93 (3.22)	3.41 (3.23)	75%	30%
M Zelen et al. [15]	2014	USA	RCT	M: 19 - F: 21	M:NA - F: NA	60.8 (10.9)	NA	NA	2.0 (1.3)	Complete healing by week 8	NA
Tettlebach et al. [16]	2019	USA	Multicenter RCT	*47	*51	57.4 (10.6)	57.1 (10.5)	3.9 (3.8)	3.2 (2.8)	At 16 weeks = 22.04%	At 16 weeks = 11.02%
M Zelen et al. [17]	2013	USA	Non-blinded RCT	M: 9 - F: 4	M: 7 - F: 5	56.4 (14.7)	61.7 (10.3)	3.4(2.9)	2.6 (1.9)	At 12 weeks = 18 patients	At 12 weeks = 5 patients
M Zelen et al. [18]	2016	USA	Multicenter RCT	M:19 - F: 13	M:22 - F: 13	63.3 (12.25)	60.6 (11.55)	1.7 (2.7)	1.7 (2.6)	31%	10%

A comprehensive analysis was conducted on a total of 633 participants enrolled across 10 RCTs [9-18]. The primary objective was to investigate the efficacy of amniotic membrane as a treatment modality. Participants were randomly assigned to either a treatment group receiving amniotic membrane (n=323) or a control group receiving standard of care (n=310).

The mean age of participants was approximately 60 years, with variability across studies. Regarding gender distribution, the majority were male (n=453) compared to female (n=180). Three studies reported comorbidities including diabetes, hypertension, heart failure, and coronary artery disease, which were present in 125 participants. All studies utilized human amniotic membrane rather than synthetic types. For ulcer etiology, diabetes was the primary cause in most studies, however, two studies did not report etiology [9,10], limiting understanding of causal factors in those cases. Mean ulcer size was 4.3 cm^2^ in the standard care group and 3.6 cm^2^ in the amniotic membrane group.

The detailed characteristics of the included studies are presented in Table 2. This table provides comprehensive information on the RCTs, including the study authors, publication year, study design, participant demographics, ulcer size, and positive results.

Patient-Reported Outcomes

Various studies assessed patient-reported outcomes to determine the effectiveness of the amniotic membrane treatment. The data revealed an impressive wound closure rate, with over 90% of the treated ulcers achieving complete closure within the observed follow-up period. This suggests a promising potential for the amniotic membrane to promote ulcer healing.

Complication Incidence and Follow-Up Duration

Despite the encouraging wound closure rate, it's crucial to note that certain participants encountered complications during treatment. One study [11] reported complications in four patients, although it didn't elaborate on their nature. In another study [12], among 52 patients, complications spanned multiple systems: seven cardiovascular, 18 integumentary, six lymphatic, two muscular, and one digestive. Separate studies [13,14] mentioned complications related to wound and target ulcer infections, though they didn't provide further details on infection types, severity, or their implications. Each study maintained a follow-up period between 12 to 16 weeks, providing a reasonable window to gauge short-term efficacy and potential complications arising from the amniotic membrane treatment.

Healing Rate

Analysis results indicate that the amniotic group experienced a notably faster wound healing rate compared to the standard of care group. Across 10 trials, the amniotic group reported 250 events, presumably faster wound healing instances, in contrast to 131 events in the standard care group. This differential underscores the accelerated healing in the amniotic group.

**Figure 2:**
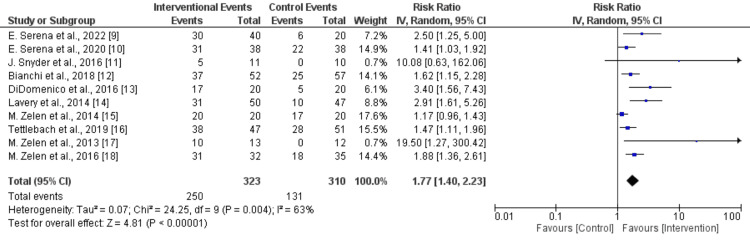
Forest plot of risk ratios for the patients who underwent management of their ulcers with human amniotic membrane dressing compared to other treatments.

A heterogeneity test revealed moderate heterogeneity with an I^2^ value of 63%. This denotes variations in treatment effects across the examined studies. The statistically significant p-value of 0.004 implies that this heterogeneity isn't merely coincidental. Thus, it hints at external factors impacting individual study results and the resultant treatment effect variation, as shown in Figure 2.

Given the moderate heterogeneity, it's imperative to interpret the findings judiciously and examine potential variation sources in the studies. Exploring subgroup or sensitivity analyses might shed light on specific factors influencing the overall outcomes. Further investigations are recommended to pinpoint and comprehend the factors responsible for the observed heterogeneity.

Risk of Bias

Two reviewers independently evaluated the risk of bias for eligible RCTs using the Cochrane Risk of Bias Assessment Tool for Randomized Trials (RoB 2). Using this revised Cochrane tool, five of the included RCTs were deemed to have a low risk of bias, while the remaining five were assessed as high risk. As per the evidence grading and recommendations from the American Society of Plastic Surgery [19], all included articles were rated as level I (Table 1).

Discussion

This systematic review and meta-analysis, grounded in 10 published RCTs, delves deep into the application and efficacy of the HAM in comparison to standard wound care for patients suffering from venous and diabetic leg ulcers. Incorporating data from 633 patients with foot ulcers (323 in the intervention group and 310 in the control group), our meta-analysis underscores the remarkable effectiveness of HAM as a treatment modality for venous and diabetic ulcers. Notably, our findings resonate with the conclusions drawn in previously published systematic reviews and meta-analyses [20,21].

The application of the amniotic membrane has proven invaluable in treating various burns and wounds. Its rich content of collagen and a plethora of growth factors enhance the healing process, thereby improving wound closure and diminishing scar formation. The amniotic membrane stands out due to its unique attributes: it lacks immunological markers, possesses antibacterial properties, and has been observed to alleviate pain upon application [22]. In a study by Zelen in 2014, the average time for complete wound healing was found to be eight weeks for the HAM group [15], which is notably quicker than most other reported RCTs. Tettlebach's research revealed that by the 12th week, 81% of wounds treated with allografts had healed, whereas in the standard of care (SOC) group, the healing rate was 55% by the same time point [16]. Such data bolster the stance that HAM not only accelerates healing time but also enhances the wound-healing process when juxtaposed with conventional treatments. Additionally, the relative risk was greater than one with a p-value less than 0.05, indicating a statistically significant positive association.

One notable observation from this meta-analysis is that a significant proportion of the participants were diabetic. Diabetic feet often exhibit complications like dysesthesia, amyotrophy, foot malformation, cracked skin, and ischemia. The toxic effects of prolonged hyperglycemia commonly precipitate neuropathy and peripheral vascular disease, making many diabetic foot ulcers (DFUs) notoriously challenging to treat. Some even progress to infection or gangrene, at times necessitating amputation to save lives [23].

The array of treatments available for venous and diabetic ulcers can be bewildering, making it challenging to determine the most effective intervention. However, as our review highlights, HAM often emerges as a superior treatment option compared to standard care.

In terms of adverse events, significant disparities in healing rates between the HAM and control groups were observed. Zelen et al.'s analysis showcased an impressive 77% wound healing rate within six weeks for the amniotic group, as opposed to a mere 8% in the standard of care group [17]. A 2019 study by Tettlebach et al. mirrored this sentiment, indicating 81% healing in the HAM group versus 55% in the control group over 12 weeks [16]. Remarkably, almost 90% of ulcers treated showcased complete closure throughout the observed period [15]. This excellent wound closure metric underscores the amniotic membrane's potential efficacy in ulcer healing. Synder et al.'s research further supports this, where 45% of patients treated with HAM achieved wound closure, contrasting starkly with 0% in the control group [11]. A few complications arose in the HAM group, encompassing cardiovascular, integumentary, lymphatic, muscular, and renal issues. However, these adverse events were fewer and not associated with the product or any study procedures, suggesting the relative safety of the treatment.

Despite the invaluable insights this systematic review offers for venous and diabetic ulcer management, there are inherent limitations. Some of these include a limited number of RCTs, small sample sizes in some studies, and a predominant elderly male patient demographic, which may influence healing times. Additionally, there was no standardized protocol for HAM preparation, potentially impacting product quality. Even though these differences seem negligible clinically, they warrant mention. Furthermore, the observation periods across trials varied between six and 16 weeks, highlighting a need to evaluate HAM's long-term efficacy and safety. In closing, future comprehensive research is needed, particularly focusing on a diverse array of cutaneous ulcers, given the majority of the studies primarily addressed diabetic ulcers and often had limited participant numbers.

## Conclusions

This systematic review and meta-analysis evaluated the utility and efficacy of HAM in managing venous and diabetic leg ulcers. The findings from the examined studies indicate that combining HAM with SOC yields superior outcomes compared to SOC alone, both in terms of healing duration and the overall wound-healing process. Notably, the majority of the studies reviewed predominantly focused on diabetic patients with leg ulcers. As such, there is a pressing need for well-designed research encompassing a broader range of cutaneous ulcers with different etiologies and a larger sample size to substantiate these findings further.
